# Fatty acid‐binding protein‐3 and renal function decline in patients with chronic coronary syndrome

**DOI:** 10.1002/clc.24210

**Published:** 2024-01-15

**Authors:** Jiunn‐Tyng Yeh, Chin‐Chou Huang, Hsin‐Bang Leu, Wei‐Hsian Yin, Wei‐Kung Tseng, Yen‐Wen Wu, Tsung‐Hsien Lin, Hung‐I Yeh, Kuan‐Cheng Chang, Ji‐Hung Wang, Chau‐Chung Wu, Jaw‐Wen Chen

**Affiliations:** ^1^ School of Medicine, College of Medicine National Yang Ming Chiao Tung University Taipei Taiwan; ^2^ Department of Medical Education Taipei Veterans General Hospital Taipei Taiwan; ^3^ Department of Medicine, Division of Cardiology Taipei Veterans General Hospital Taipei Taiwan; ^4^ Institute of Pharmacology, College of Medicine National Yang Ming Chiao Tung University Taipei Taiwan; ^5^ Cardiovascular Research Center National Yang Ming Chiao Tung University Taipei Taiwan; ^6^ Healthcare and Services Center Taipei Veterans General Hospital Taipei Taiwan; ^7^ Division of Cardiology, Heart Center Cheng‐Hsin General Hospital Taipei Taiwan; ^8^ Department of Medical Imaging and Radiological Sciences I‐Shou University Kaohsiung Taiwan; ^9^ Department of Internal Medicine, Division of Cardiology E‐Da Hospital Kaohsiung Taiwan; ^10^ Cardiology Division of Cardiovascular Medical Center Far Eastern Memorial Hospital New Taipei City Taiwan; ^11^ Division of Cardiology, Department of Internal Medicine Kaohsiung Medical University Hospital and Kaohsiung Medical University Kaohsiung Taiwan; ^12^ Mackay Medical College Mackay Memorial Hospital New Taipei City Taiwan; ^13^ Department of Internal Medicine, Division of Cardiology China Medical University Hospital Taichung Taiwan; ^14^ Graduate Institute of Clinical Medical Science China Medical University Taichung Taiwan; ^15^ Department of Cardiology, Buddhist Tzu‐Chi General Hospital Tzu‐Chi University Hualien Taiwan; ^16^ Department of Internal Medicine, Division of Cardiology National Taiwan University Hospital and National Taiwan University College of Medicine Taipei Taiwan; ^17^ Graduate Institute of Medical Education & Bioethics, College of Medicine National Taiwan University Taipei Taiwan; ^18^ Department of Medical Research and Division of Cardiology, Department of Internal Medicine Taipei Medical University Hospital Taipei Taiwan

**Keywords:** chronic coronary syndrome, coronary artery disease, estimated glomerular filtration rate, fatty acid‐binding protein 3, renal function

## Abstract

**Background:**

Renal dysfunction is common in patients with coronary artery disease. Due to the shared vascular pathogenesis between the two conditions, novel biomarkers such as the fatty acid‐binding protein‐3 (FABP‐3) have been proposed for diagnosis and prognosis prediction. This multicentre prospective cohort study investigates the association between FABP‐3 and renal dysfunction.

**Hypothesis:**

We hypothesized that higher FABP‐3 levels are correlated to worse renal outcome.

**Methods:**

Patients with chronic coronary syndrome were classified into three groups based on the initial serum FABP‐3 levels. The Chronic Kidney Disease Epidemiology Collaboration (CKD‐EPI) equation was used to estimate the patient's renal function. Renal events were defined as >25% and >50% decline in estimated glomerular filtration rate (eGFR). Cox multivariable regression was employed to delineate the correlation between FABP‐3 and renal dysfunction.

**Results:**

A total of 1606 subjects were included. During a mean follow‐up of 35.9 months, there were 239 patients with eGFR >25% reduction and 60 patients with >50% reduction. In the Kaplan–Meier survival curve and log‐rank test, increased levels of FABP‐3 were significantly correlated with eGFR >25% reduction (*p* < .001) and >50% reduction (*p* < .001). Multivariate Cox regression model revealed that subjects with higher FABP‐3 exhibited a greater risk of eGFR >25% reduction (Group 2: hazard ratio [HR] = 2.328, 95% confidence interval [CI] = 1.521–3.562, *p* < .001; Group 3: HR = 3.054, 95% CI = 1.952–4.776, *p* < .001) and >50% reduction (Group 3: HR = 4.838, 95% CI = 1.722–13.591, *p* = .003).

**Conclusions:**

Serum FABP‐3 may serve as a novel biomarker to predict eGFR decline in patients with chronic coronary syndrome.

## Graphical abstract

For patients with chronic coronary syndrome, early detection and prevention of kidney injury are crucial. We found that a higher serum FABP‐3 level was correlated to an increased risk of estimated glomerular filtration rate (eGFR) decline. Serum FABP‐3 may serve as a novel biomarker to predict eGFR decline in these patients.

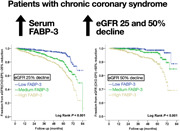

## 1 INTRODUCTION

Chronic kidney disease (CKD), defined as abnormalities of kidney structure or a glomerular filtration rate (GFR) lower than 60 mL/min/1.73 m^2^ for more than 3 months, poses a significant public health burden with a global prevalence of around 13.4%.^1^, ^2^ Despite the drastic increase in healthcare expenditure for the prevention and treatment of CKD, the number of patients suffering from end‐stage kidney disease (ESKD) is expected to increase by 29%–68% in the United States by 2030.^3^ Aside from the detrimental effect on the kidney, CKD is associated with a higher risk of cardiovascular disease (CVD).^4^ To delay the development of ESKD and improve CKD prognosis, early detection and management of CKD are crucial, especially for high‐risk populations such as CVD patients.^5^

A shared physiological mechanism underlying the pathogenesis of CKD and CVD is the disordered lipid metabolism.^6^ Recent studies have suggested that adipokines, such as the fatty acid‐binding protein (FABP) family, are involved in the modulation of lipid metabolism and have the potential to serve as biomarkers or treatment targets for a variety of disorders, such as chronic inflammation, ischemic stroke, metabolic syndrome, atherosclerosis, and CVD.^7^, ^8^ Among the FABP family, the heart‐type FABP (H‐FABP) or FABP‐3 has been considered a possible biomarker for cardiac and renal injury. FABP‐3 is a small protein expressed in cardiomyocytes and renal distal tubular cells responsible for transporting long‐chain fatty acid, which would be released to the circulation during tissue injuries, such as myocardial infarction, heart failure, and renal tubule injury.^9^, ^10^, ^11^ Also, patients with CKD are reported to have elevated levels of inflammatory and metabolic markers, such as omentin,^12^ neuregulin,^13^ serum uric acid,^14^ kidney injury molecule,^15^ prognostic nutritional index,^16^ systemic inflammatory index,^17^ uric acid/HDL‐cholesterol ratio,^18^ and C‐reactive protein,^19^ indicating correlations between inflammatory markers and renal disease. Studies have shown that the elevation of serum FABP‐3 concentration is associated with lower estimated GFR (eGFR) in type 2 diabetes mellitus (DM) patients^20^ and a higher rate for acute kidney injury after cardiac surgery.^21^ However, most of the existing studies are cross‐sectional or retrospective, which makes it difficult to infer the temporal relationship between FABP‐3 and the decline of renal function. Also, the prognostic value of FABP‐3 for kidney injury in high‐risk population such as patients with CVD is unclear.

Since the relationship between FABP‐3 and kidney injury in patients with CAD is still unclear, we aimed to examine the correlation between circulating FABP‐3 and renal function decline in patients with chronic coronary syndrome.

## 2 METHODS

### 2.1 Study design

The research is based on the “Development of New Biosignatures for Atherosclerosis Cardiovascular Diseases” study, a multicentre cohort registry that prospectively enrolled a series of patients with the chronic coronary syndrome. This study protocol has been published previously.^22^ The patients with stable coronary artery disease (CAD) after successful percutaneous coronary intervention (PCI) were included from nine tertiary referral centers in Taiwan from 2012 to 2017.

### 2.2 Patient

Subjects who fulfilled the inclusion criteria were enrolled: the presence of a significant CAD history after at least one PCI with coronary ballooning or stenting and remained clinically stable under medical treatment for at least 1 month before this enrolment. Individuals who met any of the following circumstances were excluded: under treatment with a non‐steroid anti‐inflammatory drug, steroid, disease‐modifying antirheumatic drug, or other biological immunosuppressants at enrolment or any time‐point during follow‐up, with underlying autoimmune diseases, had been hospitalized due to acute coronary syndrome in the recent 3 months, anticipated to receive coronary or other cardiac interventions in the following 1 year, undergoing therapy for compelling malignancy, mandatorily hospitalized for other systemic diseases in the following 1 year, with a life expectancy of fewer than 6 months, and failed to cooperate with clinical follow‐up.

The study complied with the principles of the Declaration of Helsinki. Approvals from each hospital's independent ethics committees and review boards were obtained (IRB: AS‐IRB01‐19007). Informed consent was obtained from all subjects before participating in this study.

### 2.3 Clinical assessment

A specially trained nurse documented demographic information following a standardized protocol from the chart or structured questionnaires. After being well‐rested, the blood pressure (BP) values were measured using an electronic BP monitor operated by a trained nurse and recorded as the average of three consecutive measurements at the outpatient clinic *ante meridiem*. Hypertension was defined as a BP level exceeding 140/90 mmHg or using antihypertensive agents. All subjects were followed on an outpatient basis at the respective institutions.

### 2.4 Biomarker measurement

Peripheral blood samples (20 cc) were collected for biochemical assessments. The samples were centrifuged before the sera were thawed for assessment. The levels of FABP‐3, as well as baseline serum chemistry, including high‐density lipoprotein‐cholesterol (HDL‐C), low‐density lipoprotein‐cholesterol (LDL‐C), and N‐terminal pro‐brain natriuretic peptide (NT‐pro‐BNP), were assessed at enrolment. Whereas renal function with creatinine was evaluated at the initial visit, every 3 months in the first year, and then at 6‐month intervals.

### 2.5 Renal events

Renal function, eGFR, was derived from serum creatinine level and demographic parameters based on Chronic Kidney Disease Epidemiology Collaboration (CKD‐EPI).^18^ Renal events were defined as a decline of over 25% and 50% from the baseline eGFR according to previous studies.^23^, ^24^, ^25^ To explore whether our results were consistent according to different equations for renal function, we further calculated eGFR based on Modification of Diet in Renal Disease (MDRD) and Cockcroft–Gault (CG) equations.^26^, ^27^, ^28^

### 2.6 Statistical analysis

Statistical Package for Social Sciences software (Version 21.0, SPSS Inc.) was used for analysis. Continuous variables were presented as mean ± SD, while categorical parameters were presented as numbers with percentages. All patients were divided into three groups (low, middle, and high) according to the FABP‐3 tertile. Parametric continuous data between the three groups were compared using a one‐way analysis of variance. Categorical data between the three groups were compared with a chi‐square test or Fisher's exact test. The Kaplan–Meier curve and log‐rank test were employed to assess the renal event rate based on FABP‐3 levels. Multivariate analysis in conjunction with the Cox proportional hazard regression model was used to evaluate the independent association between FABP‐3 levels and renal function decline. An adjustment was performed for potential confounding factors, including age, sex, BMI, systolic BP, diastolic BP, hypertension, DM, heart failure, use of antihypertensive agents, statins, baseline eGFR, HDL‐C, LDL‐C, NT‐pro‐BNP, and FABP‐3. Each parameter's hazard ratios (HRs) and 95% confidence intervals (CIs) were presented. A two‐sided *p* value less than .05 was considered statistically significant.

## 3 RESULTS

### 3.1 Baseline characteristics according to the FABP‐3 level

A total of 1606 patients with chronic coronary syndrome were included in this cohort study. The patients were categorized into three groups according to the baseline serum FABP‐3 levels. The patients with the highest FABP‐3 levels were the oldest (*p* < .001), having the highest systolic BP (*p* = .007), lowest diastolic BP (*p* = .047), highest rates of hypertension (*p* < .001), DM (*p* < .001), and heart failure (*p* < .001). They used more calcium channel blockers (*p* = .006) and diuretics (*p* < .001) but fewer statins (*p* = .002). In addition to the lowest LDL‐C levels (*p* = .018) and the highest NT‐pro‐BNP levels (*p* < .001), patients with the highest FABP‐3 levels had the highest creatinine levels (*p* < .001) and the lowest eGFR levels, which were consistent in eGFR values derived from CKD‐EPI (*p* < .001), MDRD (*p* < .001), and CG equations (*p* < .001). Detailed information on the patients is listed in Table 1.

**Table 1:** Baseline characteristics according to FABP‐3 levels.

		Group 1	Group 2	Group 3	
	All (*n* = 1606)	Low (*n* = 535)	Middle (*n* = 536)	High (*n* = 535)	*p* Value
Age, years	63.7 ± 12.0	59.8 ± 10.8	63.8 ± 11.8	67.6 ± 12.0	<.001
Male, *n* (%)	1373 (85.5%)	458 (85.6%)	460 (85.8%)	455 (85.0%)	.933
Body mass index, kg/m^2^	26.4 ± 3.8	26.0 ± 3.5	26.9 ± 3.8	26.2 ± 4.0	<.001
Systolic blood pressure, mmHg	129.9 ± 18.1	128.3 ± 16.9	129.5 ± 17.4	131.8 ± 19.9	.007
Diastolic blood pressure, mmHg	74.8 ± 12.2	75.7 ± 11.3	74.8 ± 12.4	73.9 ± 12.8	.047
Heart rate, bpm	74.3 ± 12.5	74.4 ± 11.9	73.9 ± 12.1	74.7 ± 13.5	.554
Hypertension, *n* (%)	1060 (66.0%)	322 (60.2%)	342 (63.8%)	396 (74.0%)	<.001
Diabetes mellitus, *n* (%)	595 (37.0%)	140 (26.2%)	191 (35.6%)	264 (49.3%)	<.001
Heart failure, *n* (%)	92 (5.7%)	22 (4.1%)	21 (3.9%)	49 (9.2%)	<.001
History of smoking, *n* (%)	916 (57.0%)	309 (57.8%)	308 (57.5%)	299 (55.9%)	.802
History of drinking, *n* (%)	251 (15.6%)	97 (18.1%)	82 (15.3%)	72 (13.5%)	.106
ACEI/ARB, *n* (%)	1063 (66.2%)	334 (62.4%)	361 (67.4%)	368 (68.8%)	.070
β‐Blocker, *n* (%)	1066 (66.4%)	336 (62.8%)	357 (66.6%)	373 (69.7%)	.056
CCB, *n* (%)	633 (39.4%)	192 (35.9%)	201 (37.5%)	240 (44.9%)	.006
Diuretics, *n* (%)	300 (18.7%)	56 (10.5%)	83 (15.5%)	161 (30.1%)	<.001
Antiplatelet, *n* (%)	1506 (93.8%)	506 (94.6%)	502 (93.7%)	498 (93.1%)	.594
Statins, *n* (%)	1235 (76.9%)	436 (81.5%)	412 (76.9%)	387 (72.3%)	.002
Ezetimibe, *n* (%)s	159 (9.9%)	48 (9.0%)	59 (11.0%)	52 (9.7%)	.529
Total cholesterol, mg/L	161.7 ± 37.0	163.4 ± 36.0	161.6 ± 34.8	160.2 ± 40.0	.355
Triglyceride, mg/L	136.8 ± 86.6	131.5 ± 77.3	142.3 ± 91.6	136.5 ± 90.0	.125
HDL‐C, mg/dL	41.8 ± 10.5	42.5 ± 9.9	41.8 ± 11.1	41.0 ± 10.6	.058
LDL‐C, mg/dL	93.1 ± 30.0	94.9 ± 30.6	94.2 ± 30.1	90.1 ± 29.1	.018
Cr, mg/dL	1.3 ± 1.2	1.0 ± 0.5	1.0 ± 0.4	1.8 ± 2.0	<.001
eGFR (CKD‐EPI), mL/min/1.73 m^2^	72.6 ± 24.1	85.0 ± 17.8	76.0 ± 18.6	56.8 ± 26.0	<.001
eGFR (MDRD), mL/min/1.73 m^2^	76.5 ± 30.0	89.7 ± 24.7	79.8 ± 28.8	60.0 ± 28.4	<.001
eGFR (Cockcroft–Gault), mL/min/1.73 m^2^	74.3 ± 34.4	87.9 ± 31.3	78.7 ± 33.6	56.2 ± 30.2	<.001
NT‐pro‐BNP, pg/mL	387.8 ± 872.8	269.4 ± 557.0	327.8 ± 630.9	566.2 ± 1237.3	<.001
FABP‐3, pg/mL	4455.6 ± 8969.7	1559.4 ± 410.7	2695.0 ± 356.2	9115.7 ± 14430.9	<.001

Abbreviations: ACEI, angiotensin‐converting enzyme inhibitor; ARB, angiotensin receptor blocker; CCB, calcium channel blocker; CG, Cockcroft–Gault; CKD‐EPI, Chronic Kidney Disease Epidemiology Collaboration; Cr, creatinine; eGFR, estimated glomerular filtration rate; FABP‐3, fatty‐acid‐binding proteins‐3; HDL‐C, high‐density lipoprotein‐cholesterol; LDL‐C, low‐density lipoprotein‐cholesterol; MDRD, Modification of Diet in Renal Disease; NT‐Pro‐BNP, N terminal pro B type natriuretic peptide.

### 3.2 FABP‐3 level and eGFR decline according to CKD‐EPI equation

During a mean follow‐up of 35.9 ± 23.2 months, 239 patients had eGFR >25% reduction according to CKD‐EPI equation. There were 60 patients with eGFR >50% reduction according to CKD‐EPI equation. Patients with the highest FABP‐3 levels had the most eGFR >25% reduction (*p* < .001) and >50% reduction (*p* < .001) (Table 2).

**Table 2:** Renal events according to FABP‐3 levels.

	All	Group 1	Group 2	Group 3	
	(*n* = 1606)	Low (*n* = 535)	Middle (*n* = 536)	High (*n* = 535)	*p* Value
eGFR >25% reduction, *n* (%)					
CKD‐EPI equation	239 (14.9%)	32 (6.0%)	79 (14.7%)	128 (23.9%)	<.001
MDRD equation	274 (17.1%)	52 (9.7%)	90 (16.8%)	132 (24.7%)	<.001
CG equation	286 (17.8%)	50 (9.3%)	99 (18.5%)	137 (25.6%)	<.001
eGFR >50% reduction, *n* (%)					
CKD‐EPI equation	60 (3.7%)	5 (0.9%)	15 (2.8%)	40 (7.5%)	<.001
MDRD equation	60 (3.7%)	4 (0.7%)	17 (3.2%)	39 (7.3%)	<.001
CG equation	58 (3.6%)	4 (0.7%)	16 (3.0%)	38 (7.1%)	<.001

Abbreviations: CKD‐EPI, Chronic Kidney Disease Epidemiology Collaboration; CG, Cockcroft–Gault; eGFR, estimated glomerular filtration rate; MDRD, Modification of Diet in Renal Disease.

**Figure 1:**
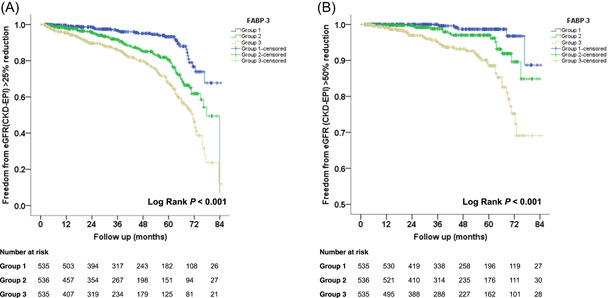
(A) Kaplan–Meier curve for >25% estimated glomerular filtration rate (eGFR) change based on the Chronic Kidney Disease Epidemiology Collaboration (CKD‐EPI) equation. (B) Kaplan–Meier curve for >50% eGFR change based on the CKD‐EPI equation.

In the Kaplan–Meier survival curve and log‐rank test, increased levels of FABP‐3 were significantly correlated with eGFR >25% reduction (*p* < .001) (Figure 1A) and >50% reduction (*p* < .001) (Figure 1B).

Multivariate analysis with the Cox regression model revealed that subjects with higher FABP‐3 exhibited a greater risk of eGFR >25% reduction (Group 2: HR = 2.328, 95% CI = 1.521–3.562, *p* < .001; Group 3: HR = 3.054, 95% CI = 1.952–4.776, *p* < .001) and >50% reduction (Group 3: HR = 4.838, 95% CI = 1.722–13.591, *p* = .003) according to the CKD‐EPI equation (Table 3).

**Table 3:** Cox regression (CKD‐EPI equation).

	eGFR > 25% reduction			eGFR > 50% reduction		
	HR	95% CI	*p* Value	HR	95% CI	*p* Value
Age, years	1.010	(0.995–0.024)	.191	1.030	(1.001–1.059)	.040
Male (yes vs. no)	0.869	(0.615–1.227)	.425	0.716	(0.356–1.440)	.349
Body mass index, kg/m^2^	0.984	(0.948–1.022)	.398	0.965	(0.895–1.040)	.345
Systolic blood pressure, mmHg	1.017	(1.007–1.026)	.001	1.022	(1.003–1.042)	.022
Diastolic blood pressure, mmHg	0.990	(0.975–1.005)	.208	0.989	(0.960–1.020)	.486
Hypertension (yes vs. no)	1.061	(0.769–1.466)	.717	0.620	(0.328–1.171)	.140
Diabetes mellitus (yes vs. no)	1.786	(1.345–2.372)	<.001	1.445	(0.816–2.561)	.207
Heart failure (yes vs. no)	0.932	(0.558–1.555)	.786	0.368	(0.093–1.449)	.153
ACEI/ARB (yes vs. no)	0.692	(0.505–0.949)	.022	1.025	(0.548–1.915)	.939
β‐Blocker (yes vs. no)	1.138	(0.847–1.530)	.390	0.667	(0.383–1.163)	.154
CCB (yes vs. no)	1.068	(0.796–1.432)	.661	1.551	(0.862–2.791)	.143
Diuretics (yes vs. no)	1.035	(0.750–1.429)	.832	0.698	(0.363–1.345)	.283
Statins (yes vs. no)	0.872	(0.637–1.192)	.390	0.743	(0.408–1.356)	.334
eGFR (CKD‐EPI), mL/min/1.73m^2^	0.999	(0.992–1.006)	.731	0.992	(0.979–1.006)	.251
HDL‐C, mg/dL	0.999	(0.986–1.013)	.921	0.981	(0.954–1.008)	.163
LDL‐C, mg/dL	1.004	(0.999–1.008)	.124	0.999	(0.990–1.009)	.907
NT‐pro‐BNP (×10^−4^), pg/mL	2.060	(1.090–3.893)	.026	1.974	(0.482–8.092)	.345
FABP‐3			<.001			.005
FABP‐3 (Group 2 vs. Group 1)	2.328	(1.521–3.562)	<.001	2.299	(0.813–6.498)	.116
FABP‐3 (Group 3 vs. Group 1)	3.054	(1.952–4.776)	<.001	4.838	(1.722–13.591)	.003

Abbreviations: ACEI, angiotensin‐converting enzyme inhibitor; ARB, angiotensin receptor blocker; CCB, calcium channel blocker; CI, confidence interval; CKD‐EPI, Chronic Kidney Disease Epidemiology Collaboration; eGFR, estimated glomerular filtration rate; FABP‐3, fatty‐acid‐binding proteins‐3; HDL‐C, high‐density lipoprotein‐cholesterol; HR, hazard ratio; LDL‐C, low‐density lipoprotein‐cholesterol; NT‐Pro‐BNP, N terminal pro B type natriuretic peptide.

### 3.3 FABP‐3 level and eGFR decline according to MDRD and CG equations

During a mean follow‐up of 35.9 ± 23.2 months, 274 and 286 patients had eGFR >25% reduction according to MDRD and CG equations, respectively. There were 60 and 58 patients with eGFR >50% reduction according to MDRD and CG equations, respectively. Patients with the highest FABP‐3 levels had the most eGFR >25% reduction (*p* < .001 for both equations) and >50% reduction (*p* < .001 for both equations) (Table 2).

In the Kaplan–Meier survival curve and log‐rank test, increased levels of FABP‐3 were significantly correlated with eGFR >25% reduction (*p* < .001 for both equations) (Figures S1A and  S2A) and >50% reduction (*p* < .001 for both equations) (Figures S1B and  S2B).

Multivariate analysis with the Cox regression model revealed that subjects with higher FABP‐3 exhibited a greater risk of eGFR (MDRD) > 25% reduction (Group 2: HR = 1.742, 95% CI = 1.222–2.483, *p* = .002; Group 3: HR = 2.643, 95% CI = 1.847–3.784, *p* < .001) and >50% reduction (Group 2: HR = 3.818, 95% CI = 1.261–11.564, *p* = .018; Group 3: HR = 9.769, 95% CI = 3.291–29.002, *p* < .001) (Table S1). Subjects with higher FABP‐3 exhibited a greater risk of eGFR (CG equation) >25% reduction (Group 2: HR = 1.981, 95% CI = 1.391–2.821, *p* < .001; Group 3: HR = 2.843, 95% CI = 1.986–4.069, *p* < .001) and >50% reduction (CG equation)(Group 2: HR = 3.399, 95% CI = 1.112–10.391, *p* = .032; Group 3: HR = 8.404, 95% CI = 2.815–25.088, *p* < .001) (Table S2).

## 4 DISCUSSION

Early detection of renal injury in high‐risked populations such as patients with CVD is crucial to prevent CKD or ESRD; however, reliable predictors or biomarkers are lacking. In this prospective cohort study, we investigated the relationship between FABP‐3 and the deterioration of renal function in patients with the chronic coronary syndrome. We found that increased serum FABP‐3 concentration is significantly associated with future eGFR reduction estimated by the three most commonly used formulas.

Also known as the heart FABP (H‐FABP), the FABP‐3 is abundantly expressed in the myocardium and is released into circulation after a cardiac injury, such as myocardial infarction.^29^ Researchers have suggested the potential of FABP‐3 as a biomarker for CVDs, including heart failure and myocardial infarction.^9^ In our cohort, albeit with wide variation, the mean serum concentration of FABP‐3 is slightly higher than that of the normal population.^30^ In combination with the fact that FABP‐3 is cleared by the kidney and reports implicating FABP‐3's renal toxicity,^21^, ^31^, ^32^ we hypothesized that the elevation of FABP‐3 in patients after cardiac damage might lead to deterioration of renal function. After correcting the baseline renal function and other potential confounders, the patient groups with higher baseline circulating FABP‐3 have significantly higher risks of the deterioration of renal function, whatever formula is used for deriving the eGFR (Table 3,  S1 and S2).

Although causality is difficult to infer from a prospective cohort study, our findings met several elements of the Bradford Hill criteria for causality, such as temporality (i.e., high FABP‐3 concentration precedes the renal event), dose–response (i.e., groups with higher FABP‐3 concentration have higher HR for the renal event), and biological plausibility.^33^ Hence, one possible mechanistic interpretation of our results is that the FABP‐3 released from the myocardial injury leads to further injury. Further studies must disentangle the relationship between FABP‐3 and cardiovascular and renal dysfunction.

From the clinical perspective, the direct implication of this study is that physicians may consider to follow FABP‐3 levels for patient with chronic coronary syndrome to assess the risk of renal damage. Early renal‐protective interventions can be initiated for high risk population to prevent the comorbidities of subsequent CKD.

The advantage of this study is the prospective design and rather big population size with detailed characterization. Regarding limitations, first, this is a non‐randomized observational study without intervention; hence the causal relationship between FABP‐3 and renal dysfunction is difficult to infer. Second, creatinine clearance was the only parameter used to assess renal function. We did not include albuminuria into our study. More metrics for renal function, such as urinary albumin excretion, may provide additional information. Third, the dynamic changes in FABP were not documented, compromising the interpretation of the longitudinal effect of the post‐CAD inflammatory burden. An extended follow‐up period is also necessary to observe the eventual prognosis of renal function impairment. Finally, contrast‐induced nephropathy might confound the interrogation of renal dysfunction after PCI. Since the study enrolled only clinically stable patients under medical treatment for at least 1 month and excluded those who anticipated receiving coronary or other cardiac interventions in the following year, such an effect is considered minimized.

## 5 CONCLUSIONS

We revealed a clear relationship between elevated FABP‐3 levels, inflammatory markers released after myocardial injury, and future decay of renal function in patients with chronic coronary syndrome. The results not only demonstrate that the FABP‐3 might serve as a predictor for the clinician to prevent the progression to CKD for these high‐risked patients but also suggest that FABP‐3 itself may be a pharmacological target for preventing renal damage.

## AUTHOR CONTRIBUTIONS

Jiunn‐Tyng Yeh and Chin‐Chou Huang conceived the research idea and established the study design. Chin‐Chou Huang, Hsin‐Bang Leu, Wei‐Hsian Yin, Wei‐Kung Tseng, Yen‐Wen Wu, Tsung‐Hsien Lin, Hung‐I Yeh, Kuan‐Cheng Chang, Ji‐Hung Wang, Chau‐Chung Wu, and Jaw‐Wen Chen were responsible for data acquisition. Chin‐Chou Huang analyzed and interpreted the data. Jiunn‐Tyng Yeh drafted the manuscript, which was revised by Chin‐Chou Huang, who offered supervision and mentorship. All authors reviewed and agreed with the final version of the article.

## CONFLICT OF INTEREST STATEMENT

The authors declare no conflict of interest.

## Supporting information


**Supporting Information**.

**Supporting Information**.

Supporting Information.

Supporting Information.

## Data Availability

Data will be available based on the reasonable request from the corresponding author.
