# Supplementary figures and images for: Fatty acid‐binding protein‐3 and renal function decline in patients with chronic coronary syndrome
Source: Clin Cardiol. 2024 Jan 15;47(1):e24210. doi: 10.1002/clc.24210 (PMC10788638; doi:10.1002/clc.24210)

Supplementary Figure 1

A

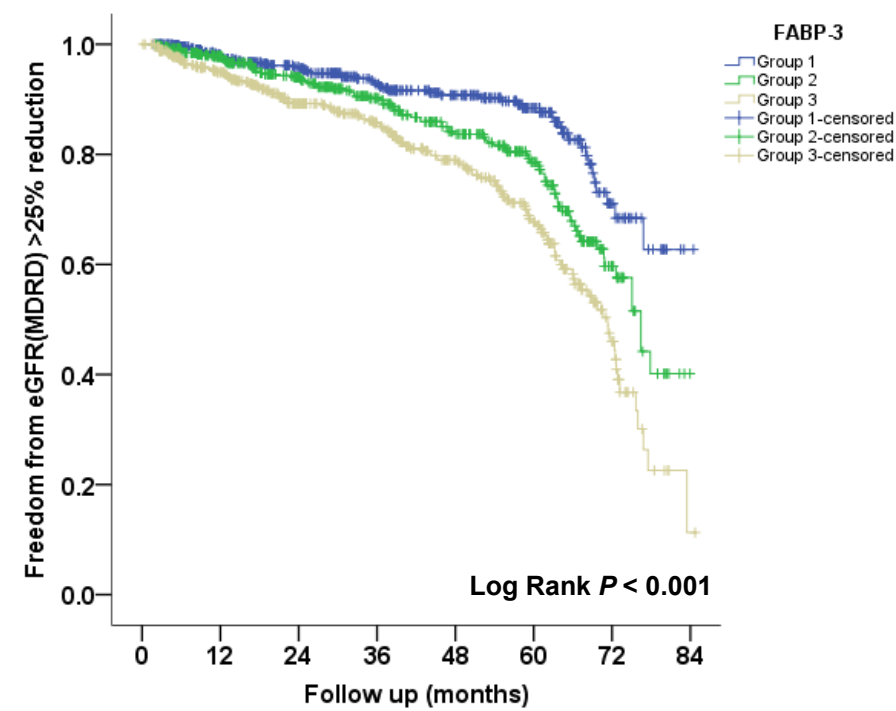

| Number at risk |     |     |     |     |     |     |     |    |
|----------------|-----|-----|-----|-----|-----|-----|-----|----|
| Group 1        | 535 | 483 | 381 | 309 | 239 | 179 | 106 | 26 |
| Group 2        | 536 | 446 | 345 | 264 | 194 | 147 | 91  | 25 |
| Group 3        | 535 | 403 | 317 | 232 | 177 | 125 | 79  | 20 |

B

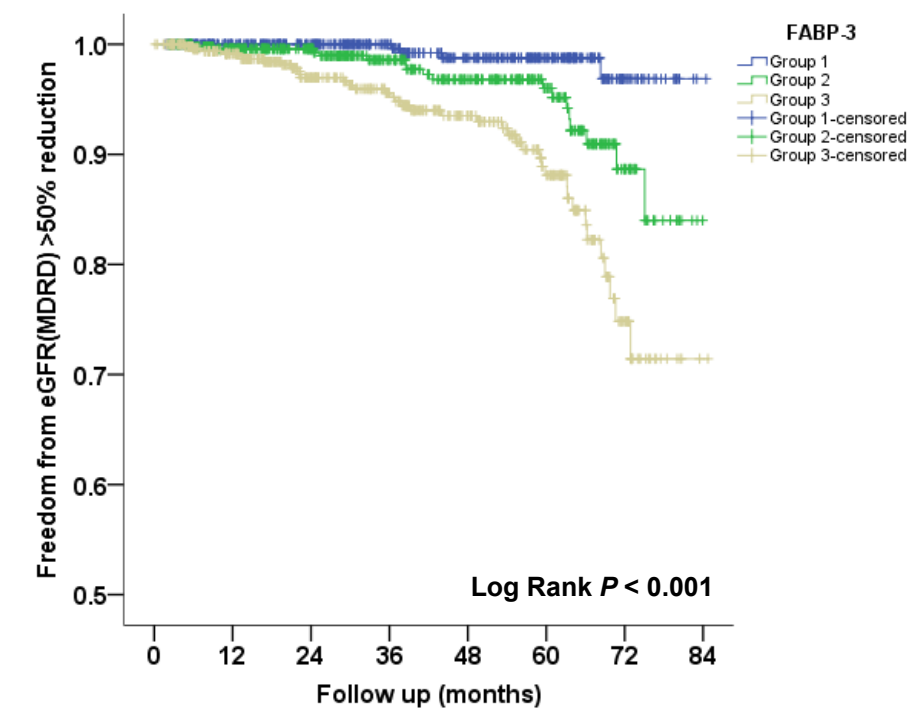

| Number at risk |     |     |     |     |     |     |     |    |
|----------------|-----|-----|-----|-----|-----|-----|-----|----|
| Group 1        | 535 | 531 | 420 | 339 | 259 | 197 | 120 | 28 |
| Group 2        | 536 | 519 | 409 | 313 | 234 | 175 | 111 | 30 |
| Group 3        | 535 | 496 | 389 | 289 | 227 | 162 | 102 | 29 |

Supplement: Supplementary file 1 — Supporting Information. [file CLC-47-e24210-s002.pdf]

Supplementary Figure 2

A

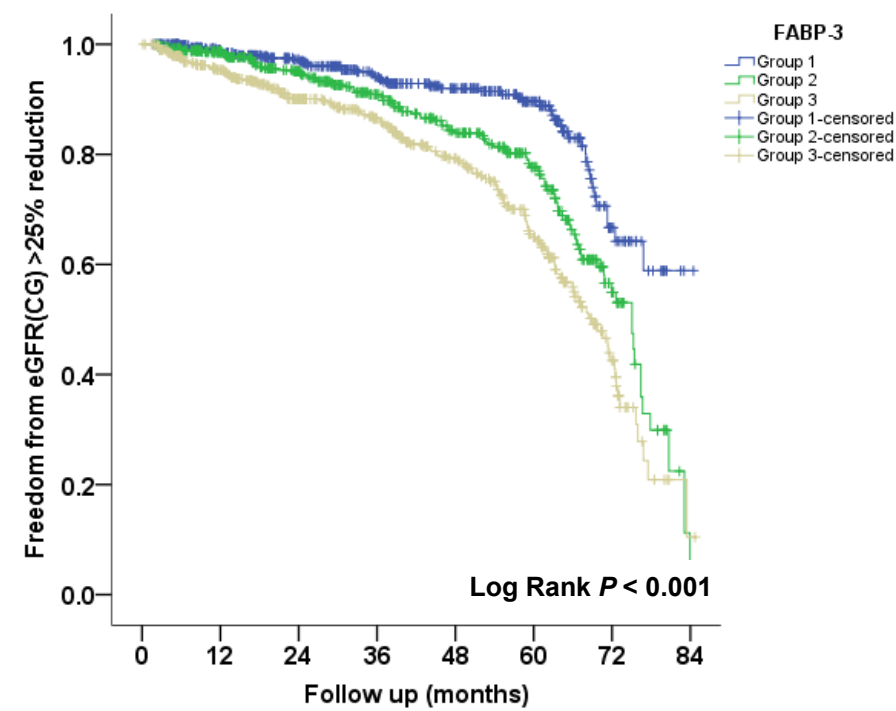

| Number at risk |     |     |     |     |     |     |     |    |
|----------------|-----|-----|-----|-----|-----|-----|-----|----|
| Group 1        | 535 | 485 | 379 | 305 | 235 | 175 | 102 | 26 |
| Group 2        | 536 | 437 | 332 | 250 | 181 | 135 | 81  | 19 |
| Group 3        | 535 | 398 | 310 | 223 | 168 | 117 | 77  | 20 |

B

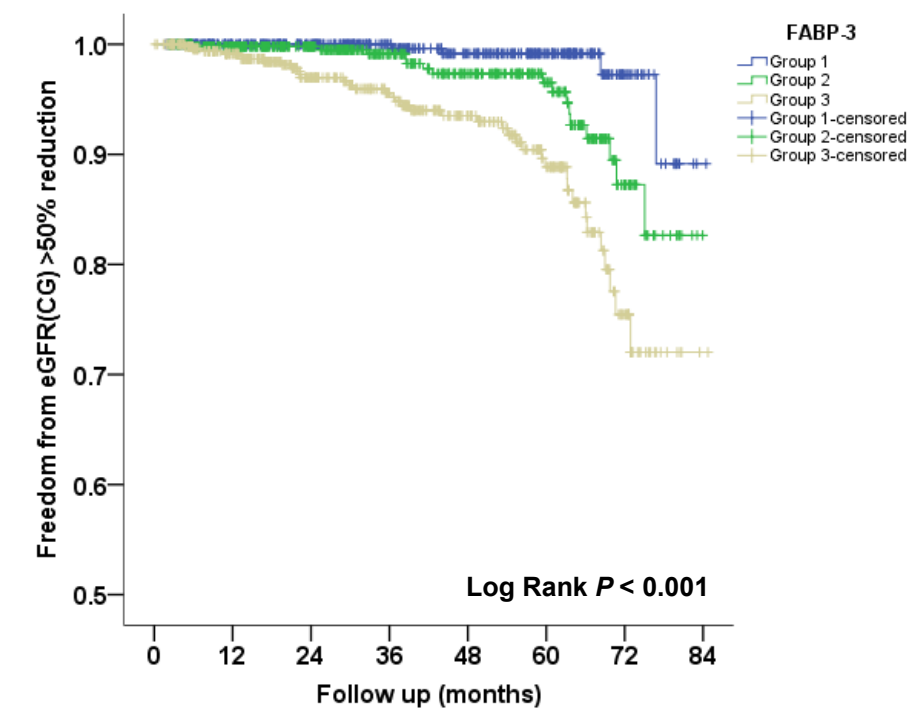

| Number at risk |     |     |     |     |     |     |     |    |
|----------------|-----|-----|-----|-----|-----|-----|-----|----|
| Group 1        | 535 | 531 | 420 | 339 | 259 | 196 | 119 | 27 |
| Group 2        | 536 | 520 | 409 | 313 | 233 | 174 | 110 | 30 |
| Group 3        | 535 | 497 | 390 | 290 | 228 | 163 | 102 | 29 |

Supplement: Supplementary file 2 — Supporting Information. [file CLC-47-e24210-s003.pdf]
