# Supplementary material for: Fatty acid‐binding protein‐3 and renal function decline in patients with chronic coronary syndrome
Source: Clin Cardiol. 2024 Jan 15;47(1):e24210. doi: 10.1002/clc.24210 (PMC10788638; doi:10.1002/clc.24210)
Supplement: Supplementary file 3 — Supporting Information. [file CLC-47-e24210-s001.docx]

**Supplementary Table 1. Cox regression (MDRD equation)**

|  | **eGFR >25% reduction** | | |  | **eGFR >50% reduction** | | |
| --- | --- | --- | --- | --- | --- | --- | --- |
|  | **HR** | **95% CI** | ***P*-value** |  | **HR** | **95% CI** | ***P*-value** |
| Age, years | 1.007 | (0.994–1.020) | 0.315 |  | 1.033 | (1.005–1.062) | 0.019 |
| Male (yes vs. no) | 0.829 | (0.600–1.144) | 0.253 |  | 0.681 | (0.342–1.359) | 0.276 |
| Body mass index, kg/m^2^ | 0.978 | (0.944–1.013) | 0.219 |  | 0.962 | (0.891–1.039) | 0.326 |
| Systolic blood pressure, mmHg | 1.020 | (1.011–1.029) | <0.001 |  | 1.025 | (1.006–1.045) | 0.010 |
| Diastolic blood pressure, mmHg | 0.987 | (0.973–1.001) | 0.067 |  | 0.993 | (0.964–1.023) | 0.644 |
| Hypertension (yes vs. no) | 0.981 | (0.731–1.317) | 0.899 |  | 0.636 | (0.339–1.194) | 0.160 |
| Diabetes mellitus (yes vs.no) | 1.670 | (1.283–2.174) | <0.001 |  | 1.597 | (0.899–2.837) | 0.110 |
| Heart failure (yes vs. no) | 1.079 | (0.675–1.723) | 0.751 |  | 0.658 | (0.207–2.088) | 0.477 |
| ACEI/ARB (yes vs. no) | 0.709 | (0.530–0.950) | 0.021 |  | 0.733 | (0.397–1.353) | 0.321 |
| β-blocker (yes vs. no) | 1.200 | (0.907–1.589) | 0.202 |  | 0.594 | (0.342–1.034) | 0.066 |
| CCB (yes vs. no) | 1.083 | (0.823–1.425) | 0.571 |  | 1.132 | (0.630–2.033) | 0.679 |
| Diuretics (yes vs. no) | 1.060 | (0.784–1.434) | 0.705 |  | 0.889 | (0.467–1.690) | 0.719 |
| Statins (yes vs. no) | 0.835 | (0.622–1.121) | 0.229 |  | 0.985 | (0.520–1.865) | 0.963 |
| eGFR (MDRD), ml/min/1.73m^2^ | 1.009 | (1.006–1.011) | <0.001 |  | 1.012 | (1.006–1.018) | <0.001 |
| HDL-C, mg/dL | 0.998 | (0.985–1.010) | 0.701 |  | 0.979 | (0.952–1.006) | 0.123 |
| LDL-C, mg/dL | 1.005 | (1.001–1.009) | 0.019 |  | 1.004 | (0.995–1.014) | 0.394 |
| NT-pro-BNP (x10^-4^), pg/mL | 2.096 | (1.116–3.935) | 0.021 |  | 1.695 | (0.415–6.929) | 0.463 |
| FABP-3 |  |  | <0.001 |  |  |  | <0.001 |
| FABP-3 (Group 2 vs. Group 1) | 1.742 | (1.222–2.483) | 0.002 |  | 3.818 | (1.261–11.564) | 0.018 |
| FABP-3 (Group 3 vs. Group 1) | 2.643 | (1.847–3.784) | <0.001 |  | 9.769 | (3.291–29.002) | <0.001 |

ACEI, angiotensin-converting enzyme inhibitor; ARB, angiotensin receptor blocker; CCB, calcium channel blocker; CI, confidence interval; eGFR, estimated glomerular filtration rate; FABP-3, fatty-acid-binding proteins-3; HDL-C, high-density lipoprotein-cholesterol; HR, hazard ratio; LDL-C, low-density lipoprotein-cholesterol; MDRD, Modification of Diet in Renal Disease; NT-Pro-BNP, N terminal pro B type natriuretic peptide.
