# Supplementary material for: Fatty acid‐binding protein‐3 and renal function decline in patients with chronic coronary syndrome
Source: Clin Cardiol. 2024 Jan 15;47(1):e24210. doi: 10.1002/clc.24210 (PMC10788638; doi:10.1002/clc.24210)
Supplement: Supplementary file 4 — Supporting Information. [file CLC-47-e24210-s004.docx]

**Supplementary Table 2. Cox regression (CG equation)**

|  | **eGFR >25% reduction** | | |  | **eGFR >50% reduction** | | |
| --- | --- | --- | --- | --- | --- | --- | --- |
|  | **HR** | **95% CI** | ***P*-value** |  | **HR** | **95% CI** | ***P*-value** |
| Age, years | 1.017 | (1.004–1.031) | 0.013 |  | 1.048 | (1.017–1.080) | 0.002 |
| Male (yes vs. no) | 0.797 | (0.579–1.097) | 0.163 |  | 0.633 | (0.314–1.276) | 0.201 |
| Body mass index, kg/m^2^ | 0.962 | (0.929–0.997) | 0.032 |  | 0.901 | (0.831–0.978) | 0.012 |
| Systolic blood pressure, mmHg | 1.018 | (1.009–1.027) | <0.001 |  | 1.023 | (1.003–1.043) | 0.027 |
| Diastolic blood pressure, mmHg | 0.989 | (0.976–1.004) | 0.145 |  | 0.992 | (0.962–1.023) | 0.617 |
| Hypertension (yes vs. no) | 0.963 | (0.721–1.286) | 0.799 |  | 0.658 | (0.344–1.259) | 0.206 |
| Diabetes mellitus (yes vs.no) | 1.535 | (1.184–1.989) | 0.001 |  | 1.568 | (0.876–2.805) | 0.130 |
| Heart failure (yes vs. no) | 1.151 | (0.730–1.814) | 0.545 |  | 0.463 | (0.119–1.809) | 0.268 |
| ACEI/ARB (yes vs. no) | 0.685 | (0.515–0.912) | 0.009 |  | 0.921 | (0.490–1.733) | 0.799 |
| β-blocker (yes vs. no) | 1.180 | (0.899–1.547) | 0.233 |  | 0.632 | (0.361–1.109) | 0.110 |
| CCB (yes vs. no) | 1.089 | (0.831–1.426) | 0.536 |  | 1.242 | (0.685–2.252) | 0.476 |
| Diuretics (yes vs. no) | 0.937 | (0.693–1.266) | 0.670 |  | 0.751 | (0.387–1.458) | 0.398 |
| Statins (yes vs. no) | 0.770 | (0.579–1.025) | 0.073 |  | 0.808 | (0.429–1.523) | 0.511 |
| eGFR (Cockcroft-Gault), ml/min/1.73m^2^ | 1.009 | (1.006–1.011) | <0.001 |  | 1.011 | (1.005–1.018) | 0.001 |
| HDL-C, mg/dL | 0.998 | (0.986–1.010) | 0.723 |  | 0.979 | (0.953–1.007) | 0.136 |
| LDL-C, mg/dL | 1.005 | (1.001–1.009) | 0.017 |  | 1.000 | (0.990–1.011) | 0.934 |
| NT-pro-BNP (x10^-4^), pg/mL | 1.789 | (0.975–3.284) | 0.060 |  | 1.844 | (0.448–7.589) | 0.397 |
| FABP-3 |  |  | <0.001 |  |  |  | <0.001 |
| FABP-3 (Group 2 vs. Group 1) | 1.981 | (1.391–2.821) | <0.001 |  | 3.399 | (1.112–10.391) | 0.032 |
| FABP-3 (Group 3 vs. Group 1) | 2.843 | (1.986–4.069) | <0.001 |  | 8.404 | (2.815–25.088) | <0.001 |

ACEI, angiotensin-converting enzyme inhibitor; ARB, angiotensin receptor blocker; CCB, calcium channel blocker; CG, Cockcroft-Gault; CI, confidence interval; eGFR, estimated glomerular filtration rate; FABP-3, fatty-acid-binding proteins-3; HDL-C, high-density lipoprotein-cholesterol; HR, hazard ratio; LDL-C, low-density lipoprotein-cholesterol; NT-Pro-BNP, N terminal pro B type natriuretic peptide.
